# Notes

**Published:** 1898-07-09

**Authors:** 


### NOTES.

The St. Patrick's Insane Asylum in Dublin, which was founded in 1750 with a bequest of £12,000 by Dean Swift for the benefit of the imbecile and insane, has just acquired a mansion surrounded by 200 acres of beautiful grounds at the suburb of Lucan, and this is to be used as a convalescent home for the asylum for the benefit of those patients who are likely to profit by change of air and scene. This is the first time such a step has been taken in connexion with an Irish insane asylum.

In connection with the present agitation to afford convalescent homes and homes for the dying for soldiers, it is interesting to learn that the Italian Government provides six convalescent homes for sick soldiers. The soldiers are said to be admirably housed in these homes, treated extremely well, and the homes are generally on Royal property, and in some cases consist of Royal palaces. In addition to such convalescent homes for the private soldier—entirely maintained by the Government—officers and non-commissioned officers are sent to various baths and medicinal springs in Italy at their country's expense, when their condition calls for such treatment. This care on the part of the Government for its convalescent soldiers is in strong contrast with our total lack of provision for such cases.

